# An unusual case of urinary tract obstruction due to imperforate hymen in an 11-month-old infant

**DOI:** 10.4103/0970-1591.32076

**Published:** 2007

**Authors:** Sajni Khemchandani, Amit Devra, Sandeep Gupta

**Affiliations:** Department of Pediatric Urology and Transplantation, Institute of Kidney Diseases and Research Center, Dr. H. L. Trivedi institute of Transplantation Sciences, Civil Hospital Campus, Ahmedabad, (Gujarat), India

**Keywords:** Female infant, imperforate hymen, urinary obstruction

## Abstract

Variations in the anatomy of the hymen are common and imperforate hymen is an extreme manifestation. In spite of the recommendations for early inspection of the external genitalia, hymenal malformations escape diagnosis until the time of menarche. Rarely, female infants with imperforate hymen present with urologic complications. We would like to present an unusual case of urinary retention in an 11-month-old female infant due to imperforate hymen. This infant was successfully treated surgically.

## CASE REPORT

**Figure 1:**
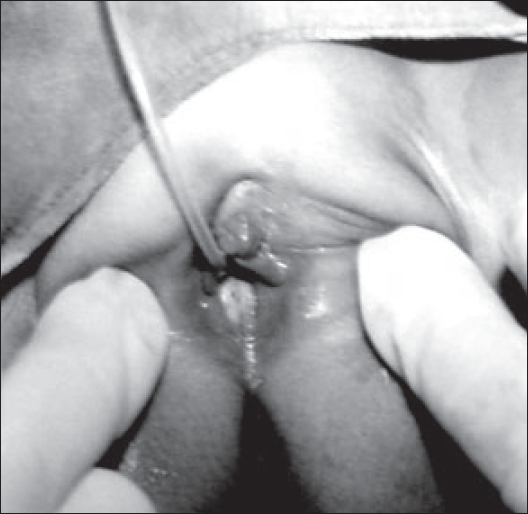
Clinical photograph showing the bulging hymen

**Figure 2:**
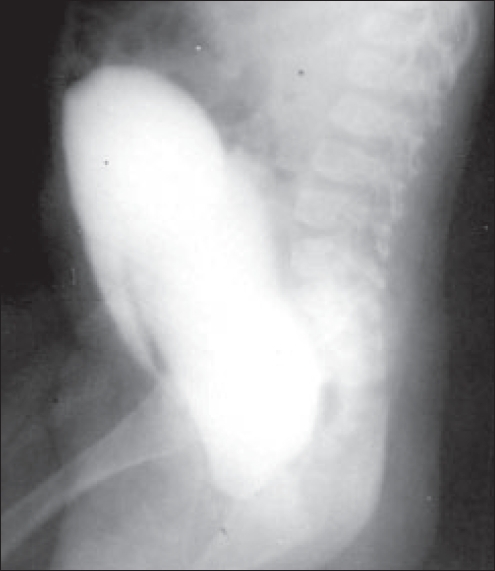
Cystourethrogram and vaginogram (lateral view)

We report an unusual case of recurrent urinary retention caused by hydrocolpos in an 11-month-old infant. An 11-month-old female presented with complaints of straining during micturition and recurrent retention of urine relieved by catheterization only. Antenatal ultrasonogaphy was not done. On general examination the child did not have any other congenital abnormality. A tender oblong lump was palpable in the hypogastrium. The external urethral meatus was normal with a pink bulge at the introitus [Figure 1]. Ultrasonography revealed a fluid-filled mass posterior to partially filled urinary bladder and no upper tract changes. Renal function tests were normal. Cysto-urethrography along with vaginography was done which showed distended vagina with stretched elongated urethra and anteriorly displaced bladder [Figure 2]. Needle aspiration yielded clear fluid from distended vagina which confirmed the diagnosis of imperforate hymen. A cruciate incision was made over the hymen and hydrocolpos was drained. No per urethral catheter was placed. Immediate postoperative period was uneventful and the patient was discharged on the second postoperative day. At two years of follow-up she is asymptomatic with normal voiding, urine microscopy, renal function and anatomy.

## DISCUSSION

Anatomic variations of the patent hymen exist, with the most common configuration having a central orifice. Imperforate hymen probably is the most frequent obstructive anomaly of the female genital tract with an incidence of 0.0014 to 0.1% of newborns.[1] The lumen of the vagina is separated from the cavity of the urogenital sinus by the hymen which is an invagination of the posterior wall of the urogenital sinus. Rupture of the hymen should occur during the perinatal period. When the hymen does not rupture, delay in the diagnosis is the rule rather than exception. Typically, the condition is not diagnosed until puberty, when the young girl presents with cyclic abdominal pain, pressure symptoms and often with an abdominal and/or pelvic mass representing a large hemato-metrocolpos. Patients can also present with urinary tract infection, recurrent urinary retention,[2] lymph edema and even dilatation of the upper urinary tracts from obstruction. Hydro-metrocolpos because of imperforate hymen has also been reported in infancy.[2] Rarely, symptoms of imperforate hymen manifest antenatally as well as in the neonatal period, requiring surgical correction for life-threatening consequences.[3] Imperforate hymen is most commonly an isolated finding as in our case, but sometimes polydactyly is associated with it as in McKusick-Kaufmann syndrome.[3] Although rare, the possible occurrence of combined anomalies (i.e. imperforate hymen and transverse vaginal septum) has been reported.[4] Careful preoperative evaluation is required to differentiate from obstructing longitudinal or transverse vaginal septa. Transperineal, transrectal and abdominal ultrasonography and MRI may be beneficial in establishing the diagnosis and determining the location and thickness of a transverse vaginal septum.

The management of hematocolpos due to imperforate hymen is drainage by incising the hymen. A cruciate incision along the diagonal diameters of the hymen, rather than anterior to posterior, avoids injury to the urethra and can be enlarged by removal of excess hymenal tissue. The hymenal ring is sutured to the vaginal epithelium (marsupalization) with fine absorbable sutures. Application of 2% lidocaine jelly provides postoperative analgesia. The incision leaves the hymenal ring intact and cures the problem with near natural residual hymen. Simple aspiration or puncture of the mucocolpos without definitive enlargement of the vaginal orifice should be discouraged to avoid recurrence and development of pyocolpos. Laser and electrocautery have also been used for incision without any added advantage but with added cost for the patient. Incomplete drainage and failure of marsupalization may result in recurrent obstruction and, potentially, in ascending pelvic infection.

## References

[1] McCann J, Wells R, Simon M, Voris J (1990). Genital findings in prepubertal girls selected for non abuse: A descriptive study. Pediatrics.

[2] Chircop R (2003). A case of retention of urine and haematocolpometra. Eur J Emerg Med.

[3] El-Messidi A, Fleming NA (2006). Congenital imperforate Hymen and its life threatening consequences in the neonatal period. J Pediatr Adolesc Gynecol.

[4] Ahmed S, Morris LL, Atkinson E (1999). Distal mucocolpos and proximal hematocolpos secondary to concurrent imperforate hymen and transverse vaginal septum. J Pediatr Surg.

